# Suicidality among child and adolescent psychiatric inpatients: time trend study comparing 2000 and 2011

**DOI:** 10.1007/s00787-019-01286-9

**Published:** 2019-02-11

**Authors:** Kim Kronström, Elina Tiiri, Elina Jokiranta-Olkoniemi, Anne Kaljonen, Andre Sourander

**Affiliations:** 1 Department of Adolescent Psychiatry, Turku University Hospital, Hospital District of Southwest Finland, Turku, Finland; 2 Research Centre for Child Psychiatry, University of Turku, Turku, Finland; 3 Department of Child Psychiatry, Turku University Hospital, Hospital District of Southwest Finland, Turku, Finland; 4 Turku Institute for Child and Youth Research, University of Turku, Turku, Finland

**Keywords:** Adolescent psychiatry, Child psychiatry, Inpatient treatment, Suicidality, Time trend study

## Abstract

Child and adolescent inpatient treatment has faced major changes since the year 2000, including shorter inpatient stays and a greater use of psychotropic drugs. This study explored changes and correlates of suicidal threats and suicide acts among inpatients, by comparing Finnish cross-sectional surveys from 2000 to 2011. A questionnaire that explored the background, diagnosis and treatment characteristics of inpatients was sent to all child and psychiatric wards in Finland. The data collection was carried out on specified days in 2000 and 2011. We received comprehensive data on 504 patients from 64/69 (93%) wards in 2000 and on 412 patients from 75/79 (95%) wards in 2011. The Spectrum of Suicidal Behaviour Scale was used to explore suicidality. The prevalence of suicidality did not change in this nationwide study: suicidal threat rates were 38% in 2000 and 37% in 2011, and suicide attempts in both years were 11%. The prevalence of suicidal acts was higher among girls and teenagers, while low general functioning, defined as Children’s Global Assessment Scale scores of under 30, was associated with both suicidal threats and acts. Violent acts were associated with both suicidal threats and acts in 2000, but not in 2011. Despite changes in treatment practices and shorter inpatient stays, the prevalence of suicidality in child and adolescent inpatient treatment remained unchanged in Finland in 2000 and 2011.

## Introduction

Suicide is one of the leading global causes of death, especially among adolescents and young adults [1]. The estimates on trends in suicide rates among young people vary across the world, from increases [2] to stable rates [3], with significant differences between individual countries. In Finland, suicides among adolescents declined by one-third during 2000 [4]. Suicidal thoughts and behaviors during childhood and adolescence are robust risk factors for adult suicidality and poor psychiatric and functional outcomes in general [5]. Population-based studies show that 12–14% of adolescents have reported suicidal thoughts at some point in their lives and 4–7% have reported suicide attempts [6, 7].

There is a clear need to study suicidality among children and adolescents in inpatient settings for several reasons. First, suicidality is one of the most common reasons for admissions to inpatient treatment and there is an increased risk of suicidal behavior during inpatient treatment [8–11]. Second, adolescents treated in inpatient settings have more risk factors for suicidality than their peers. These include depression, low self-worth, dysfunctional families, adverse childhood experiences and not feeling that they are part of their peer groups [12–14]. Third, previous suicide attempts are also major risk factors for suicidality after inpatient hospitalization [15, 16]. Growing knowledge on suicidality among inpatients can help us to develop more effective evaluations and treatment methods and lower the risks of successful suicides. In recent years, there has been growing need to develop intensive open care services as an alternative to inpatient care. These seem to be equally effective, from a clinical point of view, but are also more cost-effective [17, 18]. However, most studies on intensive open care services have excluded young people with acute suicidality [17].

There are several trends that could have had an impact on the prevalence of suicidality among child and adolescent inpatients. The expansion of child and adolescent open care psychiatry [19] provides treatment for a larger number of young patients with mental health problems [20]. If a larger proportion of suicidal children and adolescents receive adequate open care treatment, the need for hospital treatment for such patients could decrease. At the same time, the growth of open care services may improve our ability to detect suicidal children and adolescents. The growth in the number of suicidal patients that have been reported could have led to increases in referrals to hospital treatment because of suicidality.

Since the year 2000, there has been a trend toward shorter inpatient treatment stays [21–23] and this trend could also have increased the prevalence of suicidality among inpatients. Shorter inpatient treatment times could imply that children and adolescent with less acute and less severe symptoms are discharged from hospital sooner and that inpatient treatment focuses more on suicidal inpatients.

The use of psychotropic medications has also grown rapidly, both in inpatient care [24–27] and in outpatient care [28, 29], which might have led to decreasing suicidality among children and adolescents. At the same time, the diagnosis of depression has become more common in both inpatient and outpatient child and adolescent patient populations [23, 30, 31]. As depression is a major risk factor for suicidality [32], the rising prevalence of depression could have an impact on prevalence of suicidality. Thus, there are trends that may have both increased and decreased the prevalence of suicidality in child and adolescent inpatient treatment.

Information about service use can be obtained from registers and patient records, but this data lacks systematic information on suicidality. To obtain reliable information about the prevalence of suicidality, and changes in inpatient populations, we need to collect information using cross-sectional surveys with the same methodology and target populations. This paper reports the first nationwide study to examine the prevalence, risk factors and time trend changes of suicidality using two cross-sectional, nationwide samples in 2000 and 2011 that employed the same methodology.

## Materials and methods

### Procedure and subjects

The data collection for this study was carried out in 2000 and 2011 and a five-page questionnaire that explored the background, diagnostic and treatment characteristics of inpatients was circulated to all child and psychiatric wards in Finland. The psychiatrists responsible for inpatient treatment were asked to fill in a questionnaire for every inpatient up to the age of 18 years who occupied a child or adolescent psychiatric bed on the chosen study day in each of the 2 years. Day patients treated in hospital wards were classified as inpatients for the purpose of this study. In 2000, there were 69 wards that provided 547 inpatient beds treating patients who just attended during the day or stayed in the unit. The data gathered in that year covered 504 inpatients in 64/69 (93%) wards, as five wards declined to take part. In 2011, data were supplied on 412 inpatients from 75/79 (95%) wards, as four wards declined to take part.

The data in this study were based on the answers that the psychiatrists gave in 2000 and 2011 on every child or adolescent receiving relevant psychiatric treatment. The five-page questionnaire comprised 22 questions about the inpatient ward and medical and background data on the patients. Both the procedure and the questionnaire have previously been described in more detail in our previous paper [23]. This paper focuses on changes in suicidal behavior and attempts between 2000 and 2011 and how suicidality correlated with the general functioning, violence, background information and diagnostic characteristics of the psychiatric inpatients.

The chairman of the ethical committee of the University Hospital of Turku has given a statement which says that due to the methodology of this study, there was no need for application to be made to the ethical committee. The data did not include details that would enable identification of and individual patient.

### Instruments

The psychiatrist responsible for the treatment estimated the suicidality during the ongoing inpatient treatment using the Spectrum of Suicidal Behaviour Scale [8, 33], which has been widely used and found to have a high inter-rater reliability [34, 35]. The five-point scale covers: (1) no suicidal ideation or behavior, (2) suicidal ideas, (3) suicidal threats, (4) mild suicide attempts, and (5) serious suicide attempts. In this study we divided the responses into three groups for the analysis: (1) no suicidality, (2) suicidal threats, including suicidal ideas and suicidal threats, and (3) suicidal attempts, which included mild and serious suicide attempts.

The patients were diagnosed as a result of normal hospital procedures in accordance with the International Classification of Diseases-Tenth Revision (ICD-10). The psychiatrists were asked to report the one or two most important psychiatric diagnoses and both were taken into account in the analysis. This means that one patient can appear in more than one diagnostic group. For practical reasons, we have only reported diagnoses given to more than ten inpatients or 3% of the total study cohort in either 2000 or 2011. The following diagnostic groups were analyzed: depression, psychosis, mania/bipolar disorder, anxiety disorders, obsessive compulsive disorder, eating disorders, childhood affective disorder, substance use, conduct or oppositional disorder, developmental disorder, autism spectrum disorders, attachment disorder and attention-deficit hyperactivity disorder (ADHD).

The patients’ general functioning levels at the time of data collection were evaluated with the Children’s Global Assessment Scale (CGAS) [36–38], which is designed to reflect the lowest level of functioning of a child or adolescent during a specific time period. The CGAS values range from one for the most functionally impaired child to 100 for the least impaired. In the questionnaire used in this study, the CGAS scores were reported at 10-point intervals, i.e., 1–10, 11–20 and so on. For the analysis, we divided inpatients into three groups according to their CGAS scores: 0–30, 31–40 and 41–100. These cutoff scores were based on the CGAS scores that would produce clinically relevant groups. Inpatients who scored 30 or lower were, by definition, unable to function in most situations. Those with CGAS scores of 31–40 had major impairment in several areas and those who scored 41 or more had good general functioning. The level of aggression or violence displayed by the inpatients was evaluated with the six-point Spectrum of Assaultive Behaviour Scale [39]. The five-point scale covers: (1) no violent ideas or behavior, (2) violent thoughts, (3) violent threats, (4) a less serious violent act, (5) a serious violent act and (6) killing someone. For our analysis, we divided the patients into two groups: those scoring 1–3 were categorized as performing no violent acts and those scoring 4–6 were categorized as performing violent acts.

### Statistical methods

Statistically significant differences between the frequency distributions were tested with Pearson’s Chi-square test or, in the case of small frequencies, with Fisher’s exact test. The differences in nominal outcome suicidality—no suicidality, suicide threats or suicide acts—were analyzed with multinomial logistic regression analysis. The interactions of year (2000 or 2011), gender (boy or girl) and age (0–12 or 13–18 years) were tested and, if there were nearly significant interactions (*p* < 0.10), were analyzed further by dividing the data by the interactive effect. Odds ratios (OR) and 95% confidence intervals (95% CI) were calculated to quantify the significant associations. *P* values of less than 0.05 were considered significant. All statistical analyses were carried out using SAS version 9.4 (SAS, Cary, NC, USA).

## Results

### Patient characteristics and suicidality

**Fig. 1:**
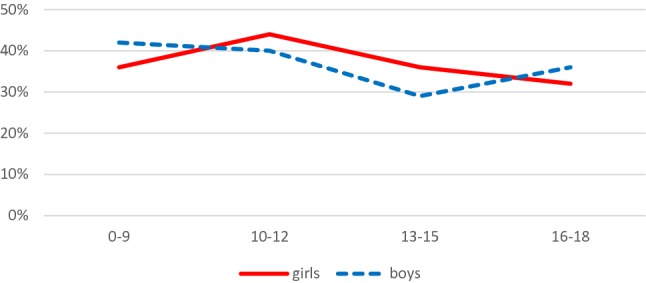
Suicidal threats among girls and boys, presented as percentages for the age groups 0–9, 10–12, 13–15 and 16–18 years

**Fig. 2:**
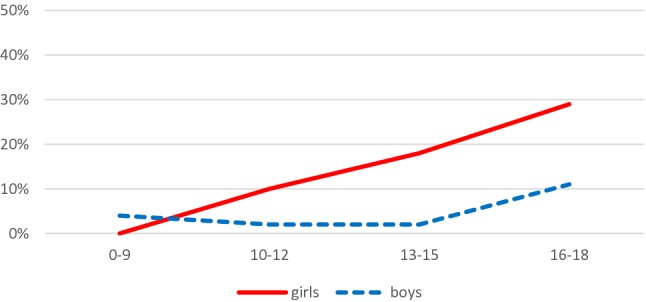
Suicidal threats among girls and boys, presented as percentages for the age groups 0–9, 10–12, 13–15 and 16–18 years

Table 1 shows that the prevalence of suicide threats at the two time points were similar, at 38% in 2000 and 37% in 2011, while the prevalence of suicidal acts were 11% in both years. In both genders, 36–38% of the inpatients made suicidal threats, whereas the prevalence of suicide acts was significantly higher among the girls than boys (19% vs. 4%, OR 6.2, 95% CI 3.7–10.5). There was no significant difference in the prevalence of suicidal threats between children (under 13 years) and teenagers (13–18 years), whereas suicidal acts were more common among teenagers (17 vs. 4%, OR 5.0, 95% CI 2.8–8.9). As shown in Figs. 1 and 2, we studied the prevalence of suicidal threats and acts separately for boys and girls in the age groups 0–9, 10–12, 13–15 and 16–18 years. The trend for suicidal acts increased strongly among girls aged 10–12 years, when compared to younger girls aged 0–9 years, and the rate of 10% was also significantly higher than the 2% recorded for boys aged 10–12 years (OR 6.2, 95% CI 1.4–27.7).

**Table 1:** The unadjusted associations, and associations adjusted by year, gender and age, between patient characteristics and suicidality (no problems/suicide threats/suicide attempts), with no suicidality as the reference group

	*N*	*P*	Unadjusted				Adjusted with year, gender and age		
			Suicidal threat		Suicidal act			Suicidal threat	Suicidal act
			*N* (%)	OR (95% CI)	*N* (%)	OR (95% CI)	*P*	OR (95% CI)	OR (95% CI)
Year		0.9580							
2000	502		189 (38)		54 (11)				
2011	409		151 (37)	1.0 (0.7–1.3)	43 (11)	1.0 (0.6–1.5)			
Gender		< 0.0001							
Boy	498		191 (38)		20 (4)				
Girl	394		142 (36)	1.2 (0.9–1.6)	76 (19)	6.2 (3.7–10.5)***			
Age (years)		< 0.0001							
0–12	406		167 (41)		15 (4)				
13–18	489		164 (34)	0.9 (0.7–1.2)	81 (17)	5.0 (2.8–8.9)***			
CGAS		0.0009					0.0046		
41–100	540		199 (37)	0.6 (0.4–1.0)*	43 (8)	0.3 (0.2–0.5)***		0.6 (0.4–1.0)*	0.3 (0.2–0.6)***
31–40	228		86 (38)	0.7 (0.4–1.1)	26 (11)	0.4 (0.2–0.8)*		0.7 (0.4–1.2)	0.5 (0.3–1.1)
0–30	109		45 (41)	1.0	22 (20)	1.0		1.0	1.0
Living with biological parents		0.4778					0.6464		
No	114		45 (39)		15 (13)				
Yes	797		295 (37)	0.8 (0.6–1.3)	82 (10)	0.7 (0.4–1.3)		0.9 (0.6–1.4)	0.7 (0.4–1.4)
Violent act			0.0027				< 0.0001		
No	657		229 (35)		63 (10)				
Yes	230		100 (43)	1.6 (1.2–2.2)**	32 (14)	1.9 (1.2–3.1)**		1.9 (1.3 -2.7)***	5.3 (2.9–9.5)***
Psychosis		0.1297					0.3929		
No	807		296 (37)		82 (10)				
Yes	104		44 (42)	1.4 (0.9–2.2)	15 (14)	1.7 (0.9–3.3)		1.4 (0.9–2.2)	1.1 (0.6–2.2)
Depression		< 0.0001					0.0021		
No	700		256 (37)		55 (8)				
Yes	211		84 (40)	1.5 (1.1–2.1)*	42 (20)	3.5 (2.2–5.6)***		1.5 (1.0–2.1)*	2.4 (1.4–3.9)***
Anxiety disorder		0.8630					0.8510		
No	816		303 (37)		86 (11)				
Yes	95		37 (39)	1.1 (0.7–1.7)	11 (12)	1.2 (0.6–2.3)		1.1 (0.7–1.8)	0.9 (0.5–2.0)
Eating disorder		0.0138					0.0262		
No	839		323 (39)		84 (10)				
Yes	72		17 (24)	0.5 (0.3–0.9)*	13 (18)	1.6 (0.8–3.1)		0.4 (0.2–0.8)**	0.6 (0.3–1.2)
Developmental disorder		0.0229					0.1748		
No	854		322 (38)		96 (11)				
Yes	57		18 (32)	0.6 (0.4–1.1)	1 (2)	0.1 (0.0–0.9)*		0.6 (0.3–1.1)	0.3 (0.0–2.2)
Autism spectrum disorder		0.0914					0.6613		
No	857		319 (37)		96 (11)				
Yes	54		21 (39)	0.9 (0.5–1.6)	1 (2)	0.1 (0.0–1.1)		1.0 (0.6–1.8)	0.4 (0.1–3.0)
ADHD		0.1274					0.5011		
No	847		319 (38)		94 (11)				
Yes	64		21 (33)	0.7 (0.4–1.2)	3 (5)	0.3 (0.1–1.1)		0.8 (0.4–1.4)	1.6 (0.4–5.8)
Conduct disorder		0.0006					0.0043		
No	692		236 (34)		83 (12)				
Yes	219		104 (47)	1.6 (1.2–2.2)**	14 (6)	0.6 (0.3–1.1)		1.8 (1.3–2.5)**	1.5 (0.8–3.0)
Emotional disorder		0.3383					0.2751		
No	829		304 (37)		91 (11)				
Yes	82		36 (44)	1.3 (0.8–2.1)	6 (7)	0.7 (0.3–1.7)		1.3 (0.8–2.1)	0.6 (0.3–1.6)

*p < 0.05; **p < 0.01;***p < 0.001

When we analyzed the combined data from 2000 to 2011, we found that suicidal acts were more prevalent among inpatients with poor general functioning, defined by a low CGAS score. While 8% of inpatients with a CGAS score of more than 41 had performed suicidal acts, the prevalence was 20% in inpatients with a CGAS score of under 30 (OR 0.3, 95% CI 0.2–0.5). There were significantly less suicidal acts among inpatients with CGAS scores of 31–40 compared to those with CGAS scores under 30 (OR 0.4, 95% CI 0.2–0.8). The association between suicidal acts and CGAS scores of 31–40 disappeared when we controlled the analysis using just the 730 “day and night inpatients” and excluded the 128 day patients treated in the hospital wards. This was the only significant association between suicidality and other variables that was incompatible between the two analyses, namely the analysis of all patients treated in the hospital wards and the analysis that only included the “day and night inpatients”.

Inpatients who committed violent acts had a higher likelihood of both suicidal threats (43% vs. 35%, OR 1.6, 95% CI 1.2–2.2) and suicidal acts (14% vs. 10%, OR 1.9, 95% CI 1.2–3.1) compared to inpatients who did not commit violent acts. The association between violence and suicidality also remained statistically significant in the adjusted multivariate model, which took into account gender, age (under or over 13 years) and the year of the study.

As presented in Table 2, we analyzed the interactions between study year, age and gender with suicidality and diagnoses. A significant interaction was found with regard to suicidality between the study year and violent acts, as violent acts were positively associated with both suicidal threats (*p* = 0.0006) and suicidal acts (*p* < 0.0001) in the year 2000, but there were no such associations in 2011. Furthermore, when the inpatients with violent acts were analyzed, we found significant decrease in suicidal acts between 2000 and 2011 (*p* = 0.002).

**Table 2:** Interactions between year, age and gender and suicidality

	*N*	*P*	Suicidal threats		Suicidal acts	
			*N* (%)	OR (95% CI)	*N* (%)	OR (95% CI)
*Act and year*						
Violent act in year 2000		< 0.001				
No	363		124 (34)		29 (8)	
Yes	135		62 (46)	2.1 (1.4–3.3)**	24 (18)	3.5 (1.9–6.6)***
Violent act in year 2011		0.5988				
No	294		105 (36)		34 (12)	
Yes	95		38 (40)	1.1 (0.7–1.9)	8 (8)	0.7 (0.3–1.7)
Eating disorder in year 2000		0.0225				
No	477		180 (38)		47 (10)	
Yes	25		9 (36)	1.4 (0.5–3.6)	7 (28)	4.1 (1.5–11.7)**
Eating disorder in year 2011		0.0155				
No	362		143 (40)		37 (10)	
Yes	47		8 (17)	0.3 (0.1–0.7)**	6 (13)	0.9 (0.4–2.3)
Autism spectrum disorder in year 2000		0.1514				
No	421		174 (41)			
Yes	27		15 (56)	1.8 (0.8–3.9)	*n* = 1, so no analysis	
Autism spectrum disorder in year 2011		0.0577				
No	340		145 (43)			
Yes	26		6 (23)	0.4 (0.2–1.0)	*n* = 1, so no analysis	
*Diagnosis and gender*						
Psychosis, boys		0.0138				
No	446		169 (38)		14 (3)	
Yes	52		22 (42)	1.4 (0.8–2.6)	6 (12)	4.7 (1.7–13.3)**
Psychosis, girls		0.7546				
No	345		122 (35)		67 (19)	
Yes	49		20 (41)	1.3 (0.7–2.5)	9 (18)	1.0 (0.5–2.4)
Depression, boys		0.9982				
No	423		162 (38)		17 (4)	
Yes	75		29 (34)	1 (0.6–1.7)	3 (4)	1.0 (0.3–3.7)
Depression, girls		< 0.0001				
No	261		88 (34)		38 (15)	
Yes	133		54 (41)	2.0 (1.2–3.3)**	38 (29)	3.3 (1.9–5.8)***

*p < 0.05; **p < 0.01;***p < 0.001

### Diagnosis and suicidality

In the analysis of the whole data, as presented in Table 1, depression was the diagnosis that was most strongly associated with suicidality. Suicidal threats (40% vs. 37%, OR 1.5, 95% CI 1.1–2.1) and suicidal acts (20% vs. 8%, OR 3.5, 95% CI 2.2–5.6) were more common among depressed inpatients than among other inpatients. The association with depression and suicidality also remained statistically significant in the adjusted model. Of the other diagnoses, having a conduct disorder was associated with suicidal threats (47% vs. 34%, OR 1.6, 95% CI 1.2–2.2) and this association remained in the adjusted model. Having a developmental disorder was associated with fewer suicidal acts (2% vs. 11% OR 0.1, 95% CI 0.0–0.9), but did not show significant associations with suicidality in the adjusted model.

When we analyzed possible interactions (Table 2), we found that in 2000 eating disorders were associated with suicidal acts (*p* = 0.0072), whereas in 2011 inpatients with eating disorders made fewer suicidal threats than other inpatients (*p* = 0.0041). There were no significant associations found between the inpatients’ age and the other variables we studied.

When interactions related to gender were studied, we found that having a psychosis was associated with a higher likelihood of suicidal acts among boys (*p* = 0.037), but not suicidality among girls. The opposite was true with regard to depression: among girls depression was associated with both suicidal threats (*p* = 0.0046) and suicidal acts (*p* < 0.0001), whereas depression was not associated with suicidality among boys.

## Discussion

The prevalence of suicidal threats and attempts among child and adolescent psychiatry inpatients remained stable in Finland from 2000 to 2011. This stability is noteworthy, because there were major changes in both inpatient and outpatient child and adolescent care in Finland during this period, as outlined earlier. In Finland, there has been a trend toward shorter hospital stays, a rapid increase in the use of psychotropic medication in inpatient and outpatient care and an increase in diagnoses of depression [11, 19, 23, 27], which could have had an impact on the prevalence of suicidality.

We found that the prevalence of suicidal threat during inpatient treatment was between 29 and 44% among both genders and across different age groups treated in child and adolescent psychiatric wards. Suicidal acts by boys during inpatient treatment were quite rare: the prevalence was less than 5% in younger age groups, but increased to 11% in the oldest age group of 16–18 years. The prevalence of suicidal acts among girls increased gradually as they got older and reached 29% in the oldest age group. The finding that suicidal acts were more common among girls and teenagers, than boys and children, agreed with previous studies [6, 40, 41]. Female teenagers generally posed a higher risk of suicidal threats and acts than males, but successful suicides were more common among males [42–44]. The association between low general functioning and suicidal threats and acts was also in line with previous findings [35].

There were associations between violent acts and both suicidal threats and acts in 2000, but no such associations were found in 2011. At the same time, the prevalence of adolescent inpatients with psychosis more than halved from 23 to 11%, as did conduct disorders, from 21 to 8% [23]. Due to the strong interrelation between violent acts and suicidality in both psychosis and conduct disorders [45, 46], it is possible the decrease of inpatients with these conditions from 2000 to 2011 made the association between violence and suicidality disappear.

Depression was associated with both suicidal threats and acts among inpatients. A further analysis showed that the association between depression and suicidality was solely down to the strong association among girls. It was somewhat surprising to us that we did not find any association between depression and suicidality among boys, as the link has been well documented in both genders [47–49]. The prevalence of suicidal threats among boys diagnosed with depression was 34% in our study, which was lower than the prevalence among inpatients boys with other diagnoses (38%). Possible explanations for the lack of any statistical association between suicidal threats and depression among boys include the high prevalence of suicidal threats among boys with other diagnoses, such as conduct disorders. Both the number of depressive inpatients and the number of suicidal acts were much smaller among boys than girls. Due to this, the analysis of the association between suicidal acts and depression among boys resulted in wide confidence intervals and the fact that we found no significant association may be due to the lack of statistical power.

Psychosis was associated with suicidal acts among boys, but there was no statistical association between psychosis and suicidality in girls. Suicidal threats were common in both genders with psychosis, but these did not reach statistical significance. It seems likely that the association between suicidality and psychosis is weaker among teenage inpatients [50] than the strong association found in other patient populations [47, 51, 52]. This may have been due to the high level of suicidal threats and acts among inpatient with other diagnoses than psychosis.

### Strengths and limitations

The main strength of this study was our very good national coverage of child and teenage inpatients, which produced a wealth of comparable data, as both of the study years used the same methodology and target groups. The population using the inpatient treatment services in our study was highly selective and there are significant differences in treatment systems between countries. Due to this, the results of this study cannot be generalized to other populations or countries. The clinicians were the only informants and the information that was received was based on their clinical evaluation, as no systematic structured interviews were performed. The main limitation of this study was the lack of operationalized criteria for scoring the questionnaire and no checks on the inter-rater reliability with regard to suicidality, violence, CGAS or diagnoses.

## Conclusion

This study showed that the prevalence of suicidal threats and suicidal acts during inpatient treatment in child and adolescent psychiatry remained stable from 2000 to 2011 in Finland. The risk of suicidal acts was particularly high in teenage girls. Although comprehensive studies on this topic are scarce, it seems likely that suicidality is also a major, ongoing challenge in child and adolescent inpatient treatment in other countries.
